# Melioidosis Presenting as a Mycotic Aneurysm of the Aortic Arch in a Patient With Pyrexia of Unknown Origin: A Case Report

**DOI:** 10.7759/cureus.109237

**Published:** 2026-05-19

**Authors:** Baker N Fenn, Krishnakumar S, Netto G Mundadan, Athul Gurudas, Juby John

**Affiliations:** 1 Infectious Diseases, Government Medical College Kottayam, Kottayam, IND

**Keywords:** aortic arch, burkholderia pseudomallei, fdg pet/ct, mycotic aneurysm, pyrexia of unknown origin

## Abstract

Melioidosis is an infection caused by the environmental saprophyte *Burkholderia pseudomallei *and is endemic to tropical regions. It has diverse clinical manifestations; however, vascular involvement in the form of a mycotic aneurysm is rare and associated with significant morbidity and mortality. We describe a 50-year-old male farmer from Kerala, India, with a nine-month history of recurrent fever, weight loss, and fatigue who underwent multiple admissions and exhaustive evaluation for pyrexia of unknown origin. Sequential blood cultures over this period identified a *Pseudomonas* species and a non-fermenting Gram-negative bacillus (NFGNB). A focal saccular outpouching at the distal aortic arch had been documented on a computed tomography scan two months before presentation to our institution, but was not initially pursued as a potential focus of infection. Fluorodeoxyglucose positron emission tomography subsequently localised abnormal uptake to this same aneurysm. Blood cultures at our institution, incubated for 10 days, grew *B. pseudomallei*. The patient became afebrile within 24 hours of initiating intravenous ceftazidime and underwent distal aortic arch replacement with a 20 mm Dacron graft and reimplantation of the subclavian arteries (7 mm each). The postoperative course was complicated by nosocomial sepsis due to carbapenem-resistant *Klebsiella pneumoniae* and ventilator-associated pneumonia, both of which resolved with targeted therapy. The patient completed eight weeks of ceftazidime-based intensive therapy and was discharged on oral cotrimoxazole eradication therapy. This case highlights several instructive issues: the recurrent misidentification of *B. pseudomallei* by conventional biochemical identification methods, the diagnostic utility of antibiogram pattern recognition, the role of fluorodeoxyglucose positron emission tomography in pyrexia of unknown origin, the importance of considering any saccular aortic aneurysm as potentially mycotic, and the limitations of applying the 2023 Duke-International Society for Cardiovascular Infectious Diseases criteria to an extracardiac vascular infection.

## Introduction

Melioidosis is a tropical bacterial infection caused by *Burkholderia pseudomallei*, a gram-negative saprophyte that lives in moist soil and surface water across South Asia, Southeast Asia, northern Australia, and parts of Africa and the Americas. Infection typically follows percutaneous inoculation, inhalation, or ingestion among people whose occupations expose them to wet earth or stagnant water. The clinical spectrum ranges from indolent skin or soft-tissue abscess to fulminant septicaemia, and almost any organ can be affected. Modelling studies estimate that India alone may account for a substantial share of the global disease burden, although under-recognition remains common [1,2].

Vascular involvement is an unusual and life-threatening manifestation. Mycotic aneurysm, defined as focal arterial wall dilatation weakened by infection, complicates an estimated 1-2% of melioidosis cases, and the aorta is the most commonly affected vessel [3,4]. Reported case series from Thailand, Singapore, Malaysia, Australia, Taiwan, China, and, more recently, the United States and Europe have together documented fewer than 100 patients, and involvement of the aortic arch is exceptionally rare, with most descriptions concerning the abdominal aorta or descending thoracic aorta [3-7].

Accurate diagnosis of melioidosis depends on isolating the organism and on laboratory familiarity with its identification. In resource-limited settings, conventional biochemical identification methods frequently misclassify *B. pseudomallei *as other non-fermenting Gram-negative bacilli (NFGNB) or as *Pseudomonas* species, particularly in regions where endemic awareness is limited, and the organism is not routinely considered in the differential. Given the recurrent and relapsing nature of melioidosis, such misidentification delays targeted therapy and may allow progression to vascular catastrophe.

We describe a patient whose diagnosis was obscured for nine months by serial microbiological misidentification, whose saccular aortic arch aneurysm was documented but initially not recognised as potentially infective, and in whom fluorodeoxyglucose (FDG) positron emission tomography with computed tomography (PET/CT) eventually localised the infective focus. We discuss the diagnostic pitfalls, the application of the 2023 Duke-International Society for Cardiovascular Infectious Diseases (ISCVID) criteria to this extracardiac infection [8], and the rationale for combined surgical and prolonged antimicrobial management.

## Case presentation

Initial presentation and prior evaluations

A 50-year-old male farmer from Kerala, India, presented with a nine-month history of intermittent fever, fatigue, and lethargy. His known comorbidities included post-traumatic epilepsy of 20 years duration, depressive illness for three years, Parkinsonism for one year, and chronic plaque psoriasis since childhood. He was not known to be diabetic or immunosuppressed.

His illness had first declared itself nine months earlier as an acute febrile episode with elevated creatinine, thrombocytopenia, and hepatic transaminitis. Leptospirosis was diagnosed on the basis of a positive IgM serology, and he recovered after a seven-day course of ceftriaxone and doxycycline. Fever recurred within weeks and prompted multiple inpatient evaluations. Laboratory work-up showed normochromic normocytic anaemia (haemoglobin 9 g/dL) with mild anisopoikilocytosis, normal total leucocyte counts and platelets, an erythrocyte sedimentation rate of 110 mm in the first hour, and C-reactive protein of 191 mg/L. Infective work-up, Widal test, blood cultures drawn at outside facilities, tuberculosis evaluation, and interferon-gamma release assay were negative. Autoimmune workup with antinuclear antibody was negative. Ultrasound of the abdomen was normal; contrast-enhanced computed tomography of the abdomen and pelvis identified only small calcific foci in the liver. Upper gastrointestinal endoscopy was performed, as he had occasional episodes of epigastric pain, suspicious of gastritis, revealed a grade II hiatus hernia with reflux oesophagitis, and colonoscopy done as part of work-up for anaemia was unremarkable.

Transthoracic echocardiography showed no vegetations. Serial blood cultures done elsewhere reported successively different organisms. A culture grew an organism reported as *Pseudomonas* species, susceptible to ceftazidime, cotrimoxazole, amoxicillin-clavulanate, and meropenem, and resistant to aminoglycosides, fluoroquinolones, and piperacillin-tazobactam; meropenem was given for seven days, and the patient was discharged on oral cotrimoxazole. During this admission, contrast-enhanced computed tomography incidentally demonstrated a focal bulge at the aortic arch near the origin of the left subclavian artery, measuring 2.7 cm by 1.9 cm. Importantly, this CT report noted no periaortic inflammatory changes and did not suggest an obvious infective aetiology; it is likely for this reason that the finding was not pursued as a potential source of infection at the time. However, in retrospect, this scan was re-assessed, and the aneurysm was noted to have wall irregularities and adjacent soft tissue thickening suggestive of peri-aortic inflammation. Two blood cultures in the subsequent month grew NFGNB with the same susceptibility profile as the previous Pseudomonas species. Over this interval, the patient received sequential courses of piperacillin-tazobactam, doxycycline, cefixime, amoxicillin-clavulanate, meropenem, and cotrimoxazole without a sustained clinical response.

In view of persistent fever with no identified source despite extensive evaluation, whole-body FDG PET/CT was performed to detect an occult infective or malignant focus. The study showed mildly increased FDG uptake localised to a saccular aneurysm of the aortic arch between the origins of the left common carotid and the left subclavian artery (maximum standardised uptake value 3.8 on early imaging and 3.4 on delayed imaging). No other FDG-avid extracardiac site was identified (Figure 1).

**Figure 1:**
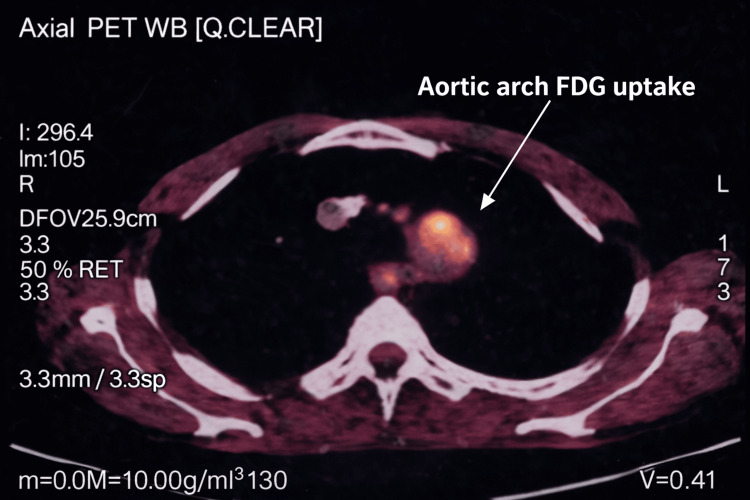
Whole-body fluorodeoxyglucose positron emission tomography with computed tomography demonstrating focal uptake within a saccular aneurysm at the distal aortic arch. No other avid focus of infection or malignancy was identified. FDG: fluorodeoxyglucose

Course at our institution

The patient was admitted to our tertiary centre for evaluation of pyrexia of unknown origin. On admission, he appeared chronically ill. Vital signs were as follows: temperature 38.2°C (recording one or two spikes per day, occasional chills, and no rigors), blood pressure 148/92 mmHg, heart rate 88 beats per minute, and respiratory rate 14 breaths per minute. Examination revealed tender palpable nodules over the pulp of the left index and middle fingers consistent with Osler nodes, grade three clubbing, psoriatic nail pitting and onycholysis, and the characteristic features of Parkinsonism (mask-like facies, slurred speech, intentional tremor, stooped posture, shuffling gait). Fundus examination was normal. There were no other peripheral stigmata of endocarditis, and no audible murmur.

Key laboratory investigations are summarised in Table 1. Rheumatoid factor was positive, and cytoplasmic antineutrophil cytoplasmic antibody (cANCA) was positive in low titre; subsequent clinical assessment found no features of granulomatosis with polyangiitis or other ANCA-associated vasculitis, and the positive cANCA was considered to reflect non-specific reactivity in the setting of chronic infection. Transoesophageal echocardiography showed no valvular vegetation. Two sets of blood cultures were drawn and incubated using conventional methods. After 10 days of incubation, both sets grew a gram-negative bacillus that was identified as *B. pseudomallei*. Susceptibility testing by disc diffusion (Kirby-Bauer method) demonstrated susceptibility to ceftazidime, imipenem, meropenem, doxycycline, tetracycline, cotrimoxazole, and amoxicillin-clavulanate, consistent with the organism’s characteristic antibiogram.

**Table 1:** Key laboratory investigations on admission to our institution ESR: erythrocyte sedimentation rate; cANCA: cytoplasmic antineutrophil cytoplasmic antibody; ANA: antinuclear antibody; RMT: rapid malaria test; ALT: alanine aminotransferase; AST: aspartate aminotransferase

Investigation	Patient value	Reference range
Haemoglobin	10 g/dL	13.0–17.0 g/dL (male)
Total leucocyte count	5,310/cumm	4,000–11,000/cumm
Neutrophils	63%	50–70%
Lymphocytes	29%	20–40%
Eosinophils	2%	1–6%
Monocytes	6%	2–8%
Platelet count	202 × 10⁹/L	150–400 × 10⁹/L
ESR	44 mm/hr (1st hour)	<15 mm/hr (male)
CRP	191 mg/L	<6 mg/L
Serum ferritin	160	12–300 ng/mL
Lactate dehydrogenase	220	140–280 U/L
Renal function (creatinine)	0.9	0.7–1.2 mg/dL
ALT	28	<40 U/L
AST	34	< 40 U/L
Rheumatoid factor	24 IU/ml	Negative (<14 IU/mL)
cANCA	Positive (low titre)	Negative
ANA	Negative	Negative
Malaria rapid test (RMT)	Negative	Negative
Blood culture ×2 (10-day incubation)	Burkholderia pseudomallei	No growth
Transoesophageal echocardiography	No vegetation	No vegetation

Intravenous ceftazidime 2 g every eight hours (150 mg/kg/day, patient weight: 40 kg) was initiated. The patient’s fever resolved within 24 hours of commencing ceftazidime. A dedicated CT angiography demonstrated saccular aneurysms arising from the superior wall of the distal aortic arch and involving the left subclavian artery at its origin, and another from an aberrant right subclavian artery just distal to its origin (Video 1, Figure 2). Given the high risk of rupture and of recurrent bacteraemia while a heavily infected vascular focus remained untreated, he was referred for surgical source control. Repeat blood cultures drawn immediately before surgery were sterile.

**Video 1:** Three-dimensional computed tomography angiography reconstruction of saccular aneurysms of the aortic arch and subclavian arteries

**Figure 2:**
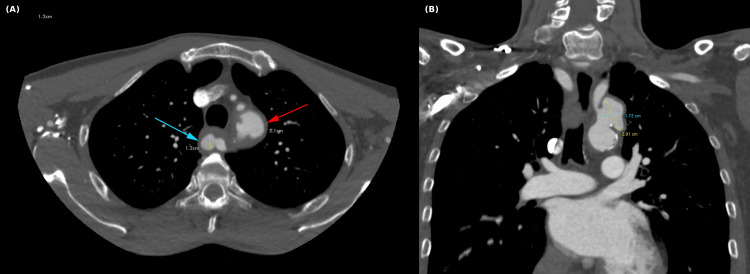
Computed tomography aortogram demonstrating two saccular aneurysms of the distal aortic arch and great vessel origins. (A) Axial section at the level of the aortic arch shows a large saccular aneurysm arising from the superior wall of the distal arch and involving the origin of the left subclavian artery (red arrow; transverse diameter 2.1 cm) and a smaller saccular outpouching arising from the aberrant right subclavian artery just distal to its origin (blue arrow; transverse diameter 1.2 cm). (B) Coronal section through the aortic arch confirms the aneurysm at the origin of the left subclavian artery (long axis 2.91 cm; short axis 1.72 cm) and demonstrates its relationship to the surrounding mediastinal structures. Both common carotid arteries arose directly from the proximal aortic arch.

Surgical management

After receiving four weeks of intravenous ceftazidime before surgery, the patient underwent distal aortic arch replacement with reimplantation of the left and aberrant right subclavian arteries under cardiopulmonary bypass. The surgical team decided to operate at four weeks of IV antibiotics as they preferred to operate in a sterile field and to reduce the risk of infecting the prosthetic Dacron graft to be implanted. As there was no evidence of imminent aneurysm rupture, this period was considered safe and appropriate. Intraoperatively, a saccular distal aortic arch aneurysm was identified at the base of the left subclavian artery and of the aberrant right subclavian artery, with both common carotid arteries arising directly from the proximal aortic arch. The distal arch was excised and replaced with a 20 mm Dacron graft. The aberrant right subclavian artery was transfixed at its origin and reconstructed with a 7 mm Dacron graft anastomosed to the ascending aorta, and the left subclavian artery was reimplanted with a further 7 mm Dacron graft (Figure 3). Ceftazidime was continued throughout the perioperative period. Aneurysm wall tissue was sent for culture, and no organism was recovered.

**Figure 3:**
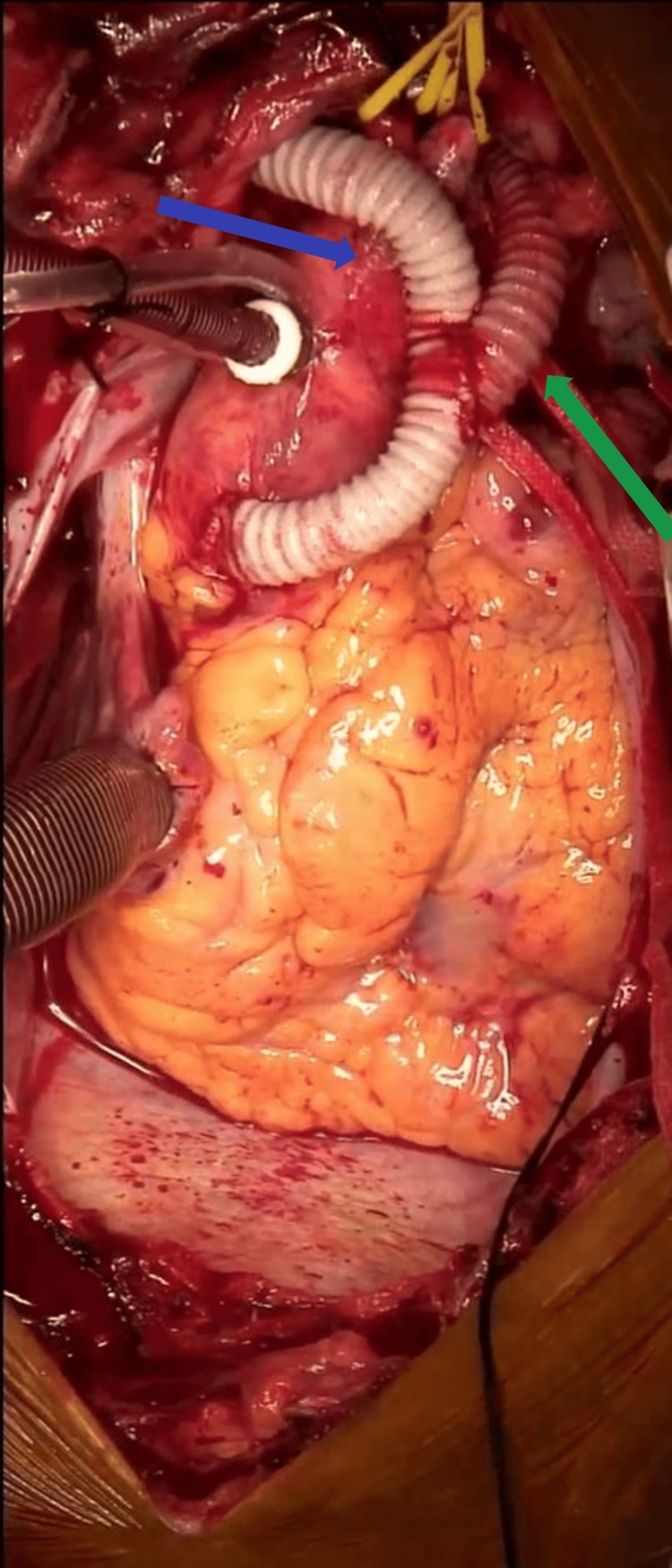
Intraoperative photograph of the repair Aberrant right subclavian artery reconstructed with a 7 mm Dacron graft (violet arrow) to the ascending aorta, and left subclavian artery reimplanted with a 7 mm Dacron graft (green arrow).

Postoperative course

The patient was extubated on the third postoperative day but required reintubation twice. On postoperative day 7, he developed new-onset fever, hypotension requiring inotropic support, and new cytopenia (total leucocyte count 1,490/cumm, platelets 130 × 10⁹/L, and haemoglobin 8 g/dL). Ceftazidime was stopped, and he was escalated to meropenem. Blood cultures grew carbapenem-resistant *Klebsiella pneumoniae* with in vitro synergy between ceftazidime-avibactam and aztreonam, and the clinical picture was consistent with a catheter-related bloodstream infection. Therapy was changed to ceftazidime-avibactam with aztreonam for 14 days. On postoperative day 14, he developed possible ventilator-acquired pneumonia, and intravenous teicoplanin was added for gram-positive cover for 12 days. He improved gradually, the tracheostomy was decannulated, all lines and drains were removed, and he was discharged afebrile in a stable condition. He received a total of eight weeks of ceftazidime-based intensive therapy (including the ceftazidime-avibactam interval used for the intercurrent CRKP bacteraemia, which was counted toward the melioidosis intensive phase as ceftazidime remained a component) and was discharged on oral cotrimoxazole 960 mg (trimethoprim 160 mg/sulfamethoxazole 800 mg) three tablets daily (12 mg/kg/day trimethoprim component) for an intended eradication phase of six months (Figure 4).

**Figure 4:**
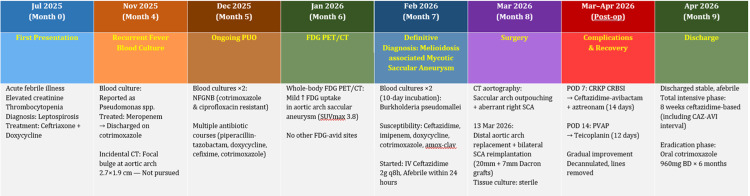
Clinical timeline NFGNB: non-fermenting gram-negative bacilli; SCA: subclavian artery; POD: postoperative day; CRKP: carbapenem-resistant Klebsiella pneumoniae; CRBSI: catheter-related bloodstream infection; PVAP: possible ventilator-acquired pneumonia; CAZ-AVI: ceftazidime-avibactam; BD: twice daily; FDG PET/CT: fluorodeoxyglucose positron emission tomography/computed tomography; SUVmax: maximum standardised uptake value; amox-clav: amoxicillin-clavulanate; Tx: treatment; CT: computed tomography

## Discussion

This case brings together several clinically instructive threads: the misidentification of *B. pseudomallei* by conventional biochemical identification methods in resource-limited laboratories, the importance of recognising that any saccular aortic aneurysm may be infective, the role of FDG PET/CT in refractory pyrexia of unknown origin, the rationale for aggressive source control in vascular melioidosis, and the difficulty of extrapolating a case of extracardiac arterial infection into the framework of the 2023 Duke-ISCVID criteria for infective endocarditis.

Melioidosis and mycotic aneurysm: a rare but highly lethal association

Mycotic aneurysm is a recognised but uncommon cardiovascular complication of melioidosis, with an estimated incidence of 1% to 2% of cases and an associated mortality in older series exceeding 50% [3,4]. Early single-institution series from Thailand, Singapore, and Australia described a predilection for elderly men with diabetes mellitus, recurrent bacteraemia, and abdominal or thoracic aortic involvement [3,4]. Subsequent Chinese, Malaysian, and North American case reports have reinforced the impression that the abdominal and descending thoracic aorta are the most frequently affected segments and that aortic arch involvement is distinctly uncommon [5-7,9,10] (Table 2). The present patient's involvement of the distal aortic arch between the origins of the great vessels is therefore unusual, and to our knowledge, only a small number of such cases have been published.

**Table 2:** Published cases of melioidosis-associated mycotic aneurysm PUO: pyrexia of unknown origin; IV: intravenous; PTFE: polytetrafluoroethylene. M: male Note: Cases represent a selective literature summary; this is not a systematic review. Some references report series rather than individual cases. The present case is the only published case of melioidosis mycotic aneurysm affecting the distal aortic arch with involvement of both subclavian arteries.

Author (year)	Age/sex	Country	Site of aneurysm	Presentation	Surgery	Antibiotic regimen	Outcome
Low et al. (2005) [3]	Multiple cases	Singapore	Aortic, femoral, popliteal (multiple sites)	Fever, septicaemia, limb pain	Yes (most)	Ceftazidime IV (intensive); cotrimoxazole (eradication)	Variable; mortality with delayed surgery
Anunnatsiri et al. (2008) [4]	59/M	Thailand	Abdominal aorta	Fever, abdominal mass	Yes	Ceftazidime + cotrimoxazole	Survived
Ding et al. (2013) [9]	53/M	Malaysia	Abdominal aorta	Fever, left iliac fossa pain radiating to back	Yes	Ceftazidime IV (intensive); cotrimoxazole + doxycycline (eradication)	Survived
Bodilsen et al. (2014) [7]	63/M	Thailand (traveller)	Abdominal Aorta	Fever, septicaemia	Yes	Meropenem IV (intensive); cotrimoxazole (eradication)	Survived
Panginikkod et al. (2017) [6]	82/M	Philippines (traveller)	Descending thoracic aorta	Fever, back pain	Yes	Ceftazidime IV (intensive); cotrimoxazole (eradication)	Survived
Wu et al. (2020) [5]	Multiple cases	China	Aorta, femoral, iliac, visceral (multiple sites)	Fever, septicaemia, local pain/mass	Yes (most)	Ceftazidime/meropenem IV; cotrimoxazole	Variable; mortality is high without surgery
Present case (2026)	50/M	India (Kerala)	Aortic arch (distal) — at the origins of the left and aberrant right subclavian arteries	Protracted PUO (9 months), recurrent bacteraemia	Yes — open distal arch replacement + bilateral subclavian reimplantation; Dacron graft	Ceftazidime IV (intensive); cotrimoxazole (eradication)	Survived; afebrile at discharge

The pathogenesis is believed to follow haematogenous seeding of a pre-existing area of endothelial injury, most often an atherosclerotic plaque, during bacteraemia. Once seeded, *B. pseudomallei* survives intracellularly within macrophages and evades clearance, producing progressive transmural destruction of the vessel wall and rapid aneurysmal dilatation [11,12]. This explains the characteristic saccular morphology of melioidosis aneurysms and their tendency to grow and rupture over weeks rather than years. More than 90% of mycotic aortic aneurysms, regardless of cause, are saccular, in contrast to degenerative atherosclerotic aneurysms, which are typically fusiform [13,14]. In practical terms, any saccular outpouching of the aorta with periaortic stranding or adjacent inflammation should be considered mycotic until proven otherwise, and this threshold is even lower in a febrile patient or one with persistently positive blood cultures. In our patient, the 2.7 cm saccular outpouching documented on earlier evaluation fulfilled exactly this description but was not flagged as a potential focus of occult infection at the time, and a further two months elapsed before the lesion was re-examined in the FDG PET/CT report.

Misidentification of *Burkholderia pseudomallei*

Perhaps the most important microbiological lesson of this case is the repeated misidentification of the causative organism before definitive isolation at our institution. Over four months, blood cultures variously labelled the organism as *Pseudomonas *species and NFGNB. In laboratories relying on conventional biochemical tests, this is a well-recognised pitfall. Organisms belonging to the NFGNB group share overlapping phenotypic characteristics, and without prior suspicion or familiarity with the organism, *B. pseudomallei, *may have been misreported as *Pseudomonas *species or simply as NFGNB using standard biochemical panels [15,16]. Even in laboratories with access to automated identification platforms, published studies report correct identification rates as low as 19% with some systems, underscoring that the problem is not confined to resource-limited settings [15].

Two practical clues helped us reconsider the earlier results retrospectively. The first was the antibiogram itself: across all three previous cultures, the isolate was consistently susceptible to ceftazidime, meropenem, and imipenem and showed a characteristic pattern of resistance to aminoglycosides, such as gentamicin and amikacin. This profile is distinctly unusual for *Pseudomonas aeruginosa *(which is typically susceptible to aminoglycosides) and is highly suggestive of *B. pseudomallei*, which is intrinsically resistant to aminoglycosides, first- and second-generation cephalosporins, while remaining susceptible to ceftazidime, carbapenems, amoxicillin-clavulanate, doxycycline, and cotrimoxazole [15]. The second clue was the requirement for prolonged incubation: our cultures became positive only after 10 days of incubation. Conventional blood culture protocols in many laboratories proceed for five to seven days before being reported negative, and in endemic regions, extending incubation to 10 to 14 days is warranted when the clinical syndrome is compatible with melioidosis. Recognising the antibiogram pattern as highly suggestive of melioidosis would have shortened several months of empirical therapy and, in our view, is an educational point worth emphasising for laboratories in endemic regions.

Fluorodeoxyglucose PET/CT in pyrexia of unknown origin

FDG PET/CT has become an established tool for evaluating pyrexia of unknown origin when first-line investigations are non-diagnostic. A systematic review and European Association of Nuclear Medicine (EANM) consensus document reported a pooled diagnostic yield of 50% to 60%, with the imaging study directly guiding further management in a substantial proportion of cases [17]. In our patient, PET/CT was requested to localise an occult infective or malignant focus after extensive conventional evaluation had proved unrevealing, and the scan correctly identified the aortic arch aneurysm as the sole site of abnormal metabolic activity. FDG uptake in an aortic outpouching, even at a modest maximum standardised uptake value, should raise a strong suspicion for a mycotic aneurysm and should trigger dedicated angiographic imaging and reassessment of blood cultures. In our patient, the PET/CT result was the turning point of a protracted diagnostic pathway and underscored the complementary relationship between anatomic and metabolic imaging when clinical ambiguity persists.

Surgical source control and antimicrobial therapy

Medical therapy alone is inadequate for a mycotic aortic aneurysm. Reported mortality with antibiotics alone approaches 50%, and published consensus and case series recommend combined surgical resection with arterial reconstruction and a prolonged antimicrobial course [3,13,18]. For melioidosis aneurysms specifically, Anunnatsiri and colleagues, and several subsequent authors, have emphasised that cure is rarely achieved without operative source control because the intracellular persistence of the organism within vascular tissue renders eradication by antibiotics alone unreliable [4,6,10,19]. Options include excision with in-situ prosthetic or autologous reconstruction, excision with extra-anatomic bypass, and endovascular stent-graft placement. A choice of open surgery was preferred over an endovascular approach for two reasons: first, endografts placed in an actively infected field carry a substantial risk of persistent infection, graft sepsis, and recurrence; second, the aneurysm involved the distal arch extending into the great vessels, making a branched endovascular approach anatomically challenging.

Antimicrobial therapy follows the two-phase model established for invasive melioidosis [18]. The intensive phase employs intravenous ceftazidime, meropenem, or imipenem for a minimum of two weeks in uncomplicated disease and for four weeks or longer in deep-seated or vascular infection. Because prosthetic graft material is assumed to remain colonised, most authorities recommend an intensive phase of at least six to eight weeks when a graft has been placed, followed by an oral eradication phase with cotrimoxazole for three to six months, and in some centres, lifelong suppression [19,20]. Our patient received eight weeks of ceftazidime-based therapy, inclusive of the ceftazidime-avibactam interval used during the intercurrent carbapenem-resistant Klebsiella pneumoniae bacteraemia, and will continue cotrimoxazole for six months with close clinical and imaging surveillance.

Does this case meet the 2023 Duke-ISCVID criteria for infective endocarditis?

The patient’s peripheral signs (Osler nodes, a positive rheumatoid factor, persistent fever, and a vascular phenomenon in the form of a mycotic aneurysm) superficially suggested infective endocarditis and raised the question of whether the 2023 Duke-ISCVID criteria might apply [8]. A careful inventory of the criteria, however, reveals that the case does not meet the threshold for definite infective endocarditis. The pathologic criterion is not satisfied, because the lesion was a distal aortic arch aneurysm rather than an ascending aortic graft with valvular involvement, and intraoperative aneurysm tissue was culture-negative. The major microbiologic criterion is not met, because B. pseudomallei is not listed among the typical pathogens in the 2023 Duke-ISCVID document and therefore requires three or more separate positive blood culture sets to qualify. Only two sets were obtained at our institution, and the preceding positive cultures at outside hospitals cannot be reattributed without molecular confirmation. The major imaging criterion is also not fulfilled: transoesophageal echocardiography was negative, and FDG uptake was in a native aortic arch aneurysm rather than in a valve, prosthetic material, or cardiac device. Four minor criteria are satisfied (fever, a vascular phenomenon, an immunologic phenomenon, and microbiologic evidence short of a major criterion); this meets the threshold for “possible infective endocarditis” at best. The clinical reality, however, is an extracardiac mycotic aneurysm. Table 3 summarises the criteria met and not met in this case.

**Table 3:** Application of the 2023 Duke-ISCVID criteria for infective endocarditis to the present case Definite IE: pathologic criteria: microorganisms identified in vegetation, cardiac tissue, or prosthetic material OR clinical criteria: two major criteria, 1 major + 3 minor criteria, 5 minor criteria. Possible IE: 1 major + 1 minor criterion, 3 minor criteria. Rejected IE: * A firm alternative diagnosis. * Lack of recurrence after <4 days of antibiotics.*No evidence of IE at surgery/autopsy after <4 days of antibiotics. *Does not meet "Possible" criteria. TEE: transoesophageal echocardiography; FDG PET/CT: fluorodeoxyglucose positron emission tomography/computed tomography; IE: infective endocarditis; *B. pseudomallei*: *Burkholderia pseudomalle*i. Criteria based on: the 2023 Duke-ISCVID criteria [8].

Criterion	Category	Were the criteria met?	Comment
Pathologic major criterion	Major	No	No surgical or autopsy evidence of intracardiac infection; aneurysm tissue culture-negative
Typical microorganism ≥2 blood culture sets	Major (microbiologic)	No	*B. pseudomallei *is not among the typical organisms listed; it requires ≥3 positive sets as a non-typical organism. Only two sets at our institution; outside cultures cannot be formally reattributed.
Echocardiography — vegetation, abscess, or prosthetic dehiscence	Major (imaging)	No	TEE negative; no cardiac structure involvement
FDG PET/CT — abnormal uptake in valve/graft/device	Major (imaging)	No	FDG uptake in a native aortic arch aneurysm; does not meet the imaging major criterion, which requires uptake at a cardiac prosthesis or endovascular graft in the ascending aorta
Surgical major criterion — intraoperative evidence of endocardial infection	Major (surgical)	No	No intracardiac infection; extracardiac arch aneurysm confirmed during surgery
Fever (>38°C)	Minor	Yes	Persistent fever 38.1°C, one to two spikes per day
Vascular phenomena	Minor	Yes	Mycotic aortic arch aneurysm (saccular morphology, FDG-avid)
Immunologic phenomena	Minor	Yes	Positive rheumatoid factor; Osler nodes on finger pulps
Microbiologic evidence (not meeting major criterion)	Minor	Yes	Repeatedly positive blood cultures for *B. pseudomallei,* an organism consistent with endovascular infection
Overall classification	—	Possible IE only	Four minor criteria met (fever, vascular, microbiologic, immunologic). Meets ≥3 minor criterion threshold for "possible IE". Clinical diagnosis is an infected (mycotic) native aortic arch aneurysm due to *B. pseudomallei*

This mismatch reflects a known structural limitation of the Duke-ISCVID framework, which is explicitly designed for intracardiac and prosthetic valve infection. Patients with purely aortic or arterial mycotic disease commonly meet several minor criteria without satisfying a major imaging or microbiological criterion, because the relevant imaging findings (valvular vegetation, paravalvular abscess, PET uptake in an ascending aortic graft) are absent. A more accurate formulation in such patients is "infected (mycotic) native aortic aneurysm due to *B. pseudomallei*," with infective endocarditis treated as a differential that was actively excluded by transoesophageal echocardiography. The 2023 Duke-ISCVID framework [8] does not include specific criteria for extracardiac endovascular infections such as mycotic aortic aneurysms; some authors have advocated for the development of dedicated diagnostic criteria for this entity, and our case supports that call [13].

Why saccular morphology matters

A recurring point in this case is that the aneurysm's saccular morphology could have been an early diagnostic clue. Degenerative atherosclerotic aortic aneurysms are overwhelmingly fusiform; more than 90% of mycotic aortic aneurysms are saccular [13,14]. Saccular aneurysms also become symptomatic and rupture at smaller diameters than fusiform aneurysms and therefore warrant earlier intervention [13,14]. The thoracic aortic imaging literature now recommends that any saccular thoracic aortic aneurysm be evaluated for an infectious aetiology, with blood cultures, inflammatory markers, and cross-sectional imaging, including FDG PET/CT when available [13,14]. Had this principle been applied earlier, when the lesion was first incidentally documented, the several-month diagnostic delay might have been avoided.

Limitations

This is a single-patient report, and we cannot be certain that the isolates previously reported by manual biochemical methods, at outside laboratories as Pseudomonas species and NFGNB, were all *B. pseudomallei*; however, the conserved antibiogram may suggest identity with the confirmed isolate, although definitive molecular confirmation or matrix-assisted laser desorption ionization-time of flight mass spectrometry (MALDI-TOFMS) was not performed. Aneurysm tissue was culture-negative, as is commonly reported when surgery is undertaken after effective antibiotics, and the overall diagnosis rests on the blood culture isolate and the consistent clinical, imaging, and operative picture. Long-term follow-up is ongoing, and the possibility of relapse remains a recognised risk with this pathogen.

## Conclusions

Melioidosis presenting as a mycotic aneurysm of the aortic arch is a rare but highly lethal manifestation that requires a high index of suspicion, prompt surgical source control, and an extended course of pathogen-directed antibiotic therapy. This case illustrates several practical lessons: that any saccular aortic aneurysm in a febrile patient should be considered potentially infective; that FDG PET/CT can be pivotal in protracted pyrexia of unknown origin by localising an occult vascular focus; and that *B. pseudomallei *is frequently misidentified by conventional biochemical methods, with the susceptibility pattern itself offering a reliable clue to the true identity of the organism.

The case also highlights the limits of applying the 2023 Duke-ISCVID framework to an extracardiac vascular infection. Broader recognition of mycotic aneurysm as a distinct clinical entity and dedicated diagnostic criteria for endovascular infection outside the heart would have helped avoid the diagnostic delay seen here.
